# (Acetone-2κ*O*){μ-6,6′-dimeth­oxy-2,2′-[propane-1,2-diylbis(nitrilo­methyl­idyne)]diphenolato-κ^8^1:2*O* ^6^,*O* ^1^,*O* ^1′^,*O* ^6′^:*O* ^1^,*N*,*N*′,*O* ^1′^}tris(nitrato-1κ^2^ *O*,*O*′)copper(II)terbium(III)

**DOI:** 10.1107/S1600536809022077

**Published:** 2009-06-17

**Authors:** Wen-Bin Sun, Peng-Fei Yan, Guang-Ming Li, Guang-Feng Hou

**Affiliations:** a Key Laboratory of Functional Inorganic Material Chemistry (HLJU), Ministry of Education, School of Chemistry and Materials Science, Heilongjiang University, Harbin 150080, People’s Republic of China; b School of Chemistry and Materials Science, Heilongjiang University, Harbin 150080, People’s Republic of China

## Abstract

In the title heteronuclear complex, [CuTb(C_19_H_20_N_2_O_4_)(NO_3_)_3_(CH_3_COCH_3_)], the Cu^II^ ion is five-coordinated by two O and two N atoms from the 6,6′-dimeth­oxy-2,2′-[propane-1,2-diylbis(nitrilo­methyl­idyne)]diphenolate ligand (*L*) and an O atom from the acetone mol­ecule in a square-pyramidal geometry. The Tb^III^ ion is ten-coordinated by six O atoms from three chelating nitrate ligands and four O atoms from the *L* ligand. In *L*, the CH_2_–CH–CH_3_ fragment is disordered over two conformations, with refined occupancies of 0.725 (11) and 0.275 (11).

## Related literature

For the copper–gadolinium and copper–praseodymium complexes of the *N*,*N*′-bis­(3-methoxy­salicyl­idene)propane-1,2-diamino ligand, see: Kara *et al.* (2000 ▶) and Sun *et al.* (2007 ▶), respectively.
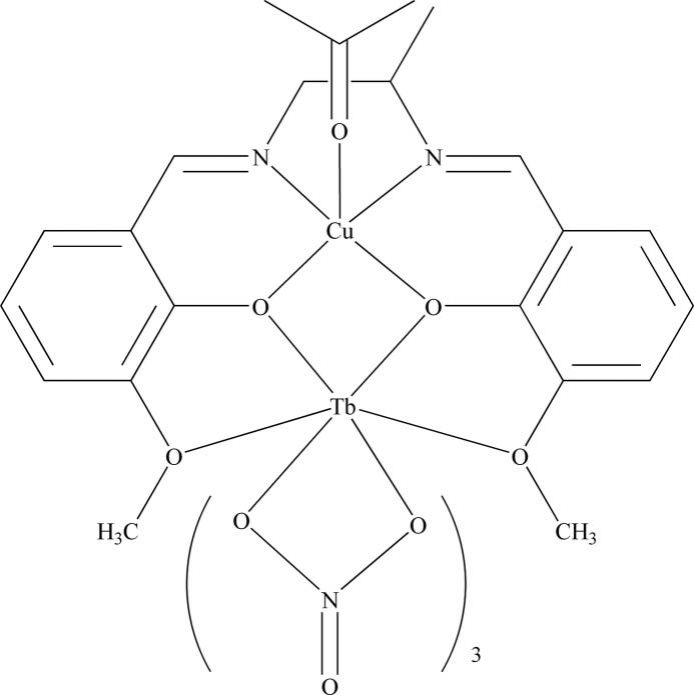


## Experimental

### 

#### Crystal data

[CuTb(C_19_H_20_N_2_O_4_)(NO_3_)_3_(C_3_H_6_O)]
*M* *_r_* = 806.94
Monoclinic, 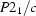

*a* = 9.8923 (9) Å
*b* = 18.8321 (18) Å
*c* = 15.5982 (15) Å
β = 95.085 (2)°
*V* = 2894.4 (5) Å^3^
*Z* = 4
Mo *K*α radiation
μ = 3.23 mm^−1^
*T* = 291 K
0.20 × 0.19 × 0.18 mm

#### Data collection

Rigaku R-AXIS RAPID diffractometer
Absorption correction: multi-scan (*ABSCOR*; Higashi, 1995 ▶) *T*_min_ = 0.563, *T*_max_ = 0.591 (expected range = 0.532–0.559)
15732 measured reflections
5696 independent reflections
4296 reflections with *I* > 2σ(*I*)
*R*_int_ = 0.037

#### Refinement

*R*[*F* ^2^ > 2σ(*F* ^2^)] = 0.040
*wR*(*F* ^2^) = 0.092
*S* = 1.02
5696 reflections
421 parameters
48 restraints
H-atom parameters constrained
Δρ_max_ = 1.12 e Å^−3^
Δρ_min_ = −0.53 e Å^−3^

### 

Data collection: *RAPID-AUTO* (Rigaku, 1998 ▶); cell refinement: *RAPID-AUTO*; data reduction: *CrystalStructure* (Rigaku/MSC, 2002 ▶); program(s) used to solve structure: *SHELXS97* (Sheldrick, 2008 ▶); program(s) used to refine structure: *SHELXL97* (Sheldrick, 2008 ▶); molecular graphics: *SHELXTL* (Sheldrick, 2008 ▶); software used to prepare material for publication: *SHELXL97*.

## Supplementary Material

Crystal structure: contains datablocks I, global. DOI: 10.1107/S1600536809022077/cv2566sup1.cif

Structure factors: contains datablocks I. DOI: 10.1107/S1600536809022077/cv2566Isup2.hkl

Additional supplementary materials:  crystallographic information; 3D view; checkCIF report

## Figures and Tables

**Fig. 1.**
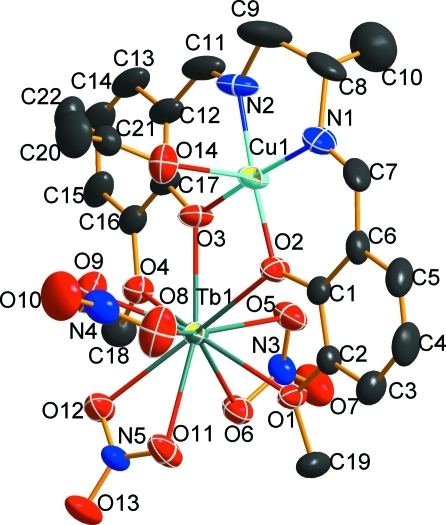
The molecular structure of the title compound showing the atomic numbering and 30% probability displacement ellipsoids. Only major part of the disordered fragment is shown. H atoms omitted for clarity.

## supplementary crystallographic information

### Comment

The Schiff base Cu—Ln (Ln = rare earth) dinuclear complexes have attracted an attention due to their magnetic properties. In the title compound (Fig. 1), the octadentate Schiff base ligand links Cu and Tb atoms into a dinuclear complex through two phenolate O atoms, that is similar with the bonding reported for other copper-gadolinium and copper-praseodymium complexes of the ligand *L* = *N*,*N*'-bis(3- Methoxysalicylidene)propane-1,2-diamine (Kara *et al.*, 2000; Sun *et al.*, 2007). The Tb^III^ centre in the title complex is ten-coordinated by four oxygen atoms from ligand *L* and six oxygen atoms from three nitrato ligands. The Cu^II^ center is five-coordinate by two nitrogen atoms and two oxygen atoms from the ligand and one oxygen atom from acetone in a square-pyramidal geometry.

### Experimental

To a 1:1 MeOH/Me_2_CO solution (20 ml) of the ligand *L* (0.086 g, 0.250 mmol) was slowly added an aqueous solution (8 ml) of [Cu(Ac)_2_H_2_O] (0.050 g, 0.25 mmol). After refluxing and stirring for 3 h, was slowly added a MeOH solution (10 ml) of Tb(NO_3_)_3_6H_2_O (0.114 g, 0.25 mmol) at ambient temperature. After stirring for 5 h, red solid was collected by filtration and washed with MeOH, [CuTb(C_19_H_20_N_2_O_4_)(CH_3_COCH_3_)(NO_3_)_3_], yield 0.172 g (85%). Single crystals suitable for X-ray determination were obtained by slow diffusion of diethylether into a methanol solution of the powder sample over one week. Analysis calculated for C_22_H_26_CuN_5_O_14_Tb: C, 32.75; H, 3.25; N, 8.68; found: C, 32.81; H, 3.30; N, 8.80%.

### Refinement

H atoms bound to C atoms were placed in calculated positions and treated as riding on their parent atoms, with C—H = 0.93 Å (aromatic C), C—H = 0.98 Å (methylene C), C—H = 0.96 Å (methly C) and with *U*_iso_(H) = 1.2Ueq(C). Atoms C8, C9 and C10 with the attached H atoms were treated as disordered over two positions with the occupancies refined to  0.725 (11) and 0.275 (11), respectively.

### Crystal data

**Table:**

[CuTb(C_19_H_20_N_2_O_4_)(NO_3_)_3_(C_3_H_6_O)]	*F*(000) = 1596
*M**_r_* = 806.94	*D*_x_ = 1.852 Mg m^−^^3^
Monoclinic, *P*2_1_/*c*	Mo *K*α radiation, λ = 0.71073 Å
Hall symbol: -P 2ybc	Cell parameters from 3804 reflections
*a* = 9.8923 (9) Å	θ = 2.3–21.9°
*b* = 18.8321 (18) Å	µ = 3.23 mm^−^^1^
*c* = 15.5982 (15) Å	*T* = 291 K
β = 95.085 (2)°	Block, red
*V* = 2894.4 (5) Å^3^	0.20 × 0.19 × 0.18 mm
*Z* = 4	

### Data collection

**Table:**

Rigaku R-AXIS RAPID diffractometer	5696 independent reflections
Radiation source: fine-focus sealed tube	4296 reflections with *I* > 2σ(*I*)
graphite	*R*_int_ = 0.037
ω scans	θ_max_ = 26.1°, θ_min_ = 1.7°
Absorption correction: multi-scan (*ABSCOR*; Higashi, 1995)	*h* = −12→12
*T*_min_ = 0.563, *T*_max_ = 0.591	*k* = −23→16
15732 measured reflections	*l* = −19→15

### Refinement

**Table:**

Refinement on *F*^2^	Primary atom site location: structure-invariant direct methods
Least-squares matrix: full	Secondary atom site location: difference Fourier map
*R*[*F*^2^ > 2σ(*F*^2^)] = 0.040	Hydrogen site location: inferred from neighbouring sites
*wR*(*F*^2^) = 0.092	H-atom parameters constrained
*S* = 1.02	*w* = 1/[σ^2^(*F*_o_^2^) + (0.0427*P*)^2^ + 0.3818*P*] where *P* = (*F*_o_^2^ + 2*F*_c_^2^)/3
5696 reflections	(Δ/σ)_max_ = 0.003
421 parameters	Δρ_max_ = 1.12 e Å^−^^3^
48 restraints	Δρ_min_ = −0.53 e Å^−^^3^

### Special details

**Table:**

Geometry. All e.s.d.'s (except the e.s.d. in the dihedral angle between two l.s. planes) are estimated using the full covariance matrix. The cell e.s.d.'s are taken into account individually in the estimation of e.s.d.'s in distances, angles and torsion angles; correlations between e.s.d.'s in cell parameters are only used when they are defined by crystal symmetry. An approximate (isotropic) treatment of cell e.s.d.'s is used for estimating e.s.d.'s involving l.s. planes.
Refinement. Refinement of *F*^2^ against ALL reflections. The weighted *R*-factor *wR* and goodness of fit *S* are based on *F*^2^, conventional *R*-factors *R* are based on *F*, with *F* set to zero for negative *F*^2^. The threshold expression of *F*^2^ > σ(*F*^2^) is used only for calculating *R*-factors(gt) *etc*. and is not relevant to the choice of reflections for refinement. *R*-factors based on *F*^2^ are statistically about twice as large as those based on *F*, and *R*- factors based on ALL data will be even larger.

### Fractional atomic coordinates and isotropic or equivalent isotropic displacement parameters (Å^2^)

**Table:**

	*x*	*y*	*z*	*U*_iso_*/*U*_eq_	Occ. (<1)
Tb1	0.28678 (3)	0.074476 (12)	0.751003 (15)	0.04448 (10)	
Cu1	0.20813 (8)	0.24090 (4)	0.67475 (5)	0.0633 (2)	
N1	0.2390 (6)	0.3365 (3)	0.7107 (4)	0.0942 (19)	
N2	0.1210 (6)	0.2826 (3)	0.5712 (4)	0.0845 (17)	
N3	0.5440 (5)	0.0470 (3)	0.6857 (3)	0.0578 (12)	
N4	0.0279 (7)	0.0890 (3)	0.8196 (4)	0.0816 (17)	
N5	0.2699 (6)	−0.0635 (2)	0.8377 (3)	0.0685 (14)	
O1	0.4538 (4)	0.12109 (18)	0.8774 (2)	0.0554 (9)	
O2	0.3134 (4)	0.19791 (18)	0.7687 (2)	0.0580 (10)	
O3	0.1984 (4)	0.14580 (18)	0.6349 (2)	0.0586 (10)	
O4	0.2368 (4)	0.01506 (19)	0.6000 (2)	0.0560 (9)	
O5	0.4863 (4)	0.1047 (2)	0.6734 (3)	0.0729 (12)	
O6	0.4893 (4)	0.0038 (2)	0.7354 (3)	0.0671 (11)	
O7	0.6477 (5)	0.0309 (3)	0.6551 (4)	0.1129 (18)	
O8	0.1350 (6)	0.1106 (3)	0.8570 (3)	0.1117 (19)	
O9	0.0401 (5)	0.0624 (3)	0.7478 (3)	0.0940 (15)	
O10	−0.0824 (6)	0.0945 (3)	0.8491 (4)	0.130 (2)	
O11	0.3161 (5)	−0.0093 (2)	0.8754 (3)	0.0913 (15)	
O12	0.2238 (5)	−0.0545 (2)	0.7621 (3)	0.0768 (13)	
O13	0.2657 (6)	−0.1202 (2)	0.8738 (3)	0.1142 (19)	
C19	0.5588 (7)	0.0802 (3)	0.9243 (4)	0.080 (2)	
H1A	0.6425	0.1062	0.9276	0.120*	
H1B	0.5700	0.0359	0.8953	0.120*	
H1C	0.5338	0.0711	0.9814	0.120*	
C2	0.4457 (5)	0.1926 (3)	0.8984 (3)	0.0519 (13)	
C3	0.5042 (6)	0.2224 (3)	0.9726 (4)	0.0738 (18)	
H1	0.5525	0.1948	1.0143	0.089*	
C4	0.4894 (7)	0.2961 (4)	0.9841 (5)	0.088 (2)	
H2	0.5261	0.3173	1.0347	0.106*	
C5	0.4232 (6)	0.3357 (3)	0.9229 (5)	0.084 (2)	
H3	0.4171	0.3844	0.9319	0.101*	
C6	0.3635 (6)	0.3078 (3)	0.8468 (4)	0.0629 (16)	
C1	0.3703 (5)	0.2330 (3)	0.8362 (4)	0.0512 (13)	
C7	0.3023 (6)	0.3546 (3)	0.7814 (6)	0.088 (2)	
H4	0.3102	0.4030	0.7922	0.106*	
C8	0.1969 (14)	0.3843 (6)	0.6358 (7)	0.098 (4)	0.688 (12)
H8	0.1681	0.4294	0.6598	0.118*	0.688 (12)
C9	0.0831 (18)	0.3580 (4)	0.5792 (11)	0.159 (10)	0.688 (12)
H9A	0.0767	0.3819	0.5239	0.191*	0.688 (12)
H9B	−0.0020	0.3632	0.6050	0.191*	0.688 (12)
C10	0.2949 (16)	0.4011 (8)	0.5767 (10)	0.165 (7)	0.688 (12)
H10A	0.3065	0.3610	0.5401	0.247*	0.688 (12)
H10B	0.3799	0.4125	0.6081	0.247*	0.688 (12)
H10C	0.2638	0.4411	0.5422	0.247*	0.688 (12)
C8'	0.128 (2)	0.3834 (11)	0.6722 (16)	0.086 (6)	0.312 (12)
H8'	0.0445	0.3815	0.7011	0.104*	0.312 (12)
C9'	0.114 (4)	0.3599 (6)	0.5828 (18)	0.093 (7)	0.312 (12)
H9'1	0.1845	0.3819	0.5527	0.112*	0.312 (12)
H9'2	0.0272	0.3766	0.5563	0.112*	0.312 (12)
C10'	0.160 (3)	0.4509 (17)	0.642 (2)	0.145 (13)	0.312 (12)
H10D	0.1263	0.4866	0.6781	0.217*	0.312 (12)
H10E	0.1187	0.4568	0.5840	0.217*	0.312 (12)
H10F	0.2566	0.4556	0.6418	0.217*	0.312 (12)
C11	0.0870 (7)	0.2485 (5)	0.5039 (5)	0.090 (2)	
H5	0.0502	0.2748	0.4570	0.108*	
C12	0.0984 (6)	0.1733 (4)	0.4908 (4)	0.0711 (18)	
C13	0.0550 (7)	0.1448 (5)	0.4103 (4)	0.088 (2)	
H6	0.0158	0.1747	0.3676	0.105*	
C14	0.0686 (7)	0.0750 (5)	0.3928 (4)	0.093 (3)	
H7	0.0362	0.0576	0.3391	0.112*	
C15	0.1290 (6)	0.0298 (4)	0.4528 (3)	0.0719 (18)	
H15	0.1404	−0.0179	0.4397	0.086*	
C16	0.1730 (5)	0.0556 (3)	0.5332 (3)	0.0553 (14)	
C17	0.1551 (5)	0.1262 (3)	0.5541 (3)	0.0555 (14)	
C18	0.2845 (7)	−0.0536 (3)	0.5759 (4)	0.0720 (17)	
H18A	0.2083	−0.0841	0.5608	0.108*	
H18B	0.3397	−0.0738	0.6234	0.108*	
H18C	0.3372	−0.0487	0.5275	0.108*	
O14	−0.0246 (4)	0.2439 (2)	0.7447 (3)	0.0792 (13)	
C20	−0.2527 (8)	0.2460 (5)	0.7791 (5)	0.113 (3)	
H20A	−0.2683	0.2062	0.8154	0.169*	
H20B	−0.3344	0.2568	0.7437	0.169*	
H20C	−0.2263	0.2864	0.8143	0.169*	
C21	−0.1421 (7)	0.2283 (3)	0.7233 (4)	0.0689 (17)	
C22	−0.1803 (7)	0.1933 (4)	0.6404 (4)	0.090 (2)	
H22A	−0.1000	0.1768	0.6162	0.135*	
H22B	−0.2272	0.2265	0.6017	0.135*	
H22C	−0.2385	0.1537	0.6492	0.135*	

### Atomic displacement parameters (Å^2^)

**Table:**

	*U*^11^	*U*^22^	*U*^33^	*U*^12^	*U*^13^	*U*^23^
Tb1	0.05575 (17)	0.03633 (15)	0.04035 (16)	0.00036 (12)	−0.00140 (11)	0.00182 (11)
Cu1	0.0784 (5)	0.0434 (4)	0.0669 (5)	0.0103 (3)	0.0002 (4)	0.0158 (3)
N1	0.104 (5)	0.044 (3)	0.128 (5)	0.001 (3)	−0.023 (4)	0.018 (3)
N2	0.104 (4)	0.077 (4)	0.073 (4)	0.034 (3)	0.011 (3)	0.037 (3)
N3	0.058 (3)	0.047 (3)	0.071 (3)	0.001 (2)	0.021 (3)	−0.004 (3)
N4	0.088 (5)	0.073 (4)	0.088 (5)	0.007 (3)	0.032 (4)	0.006 (3)
N5	0.091 (4)	0.043 (3)	0.068 (4)	−0.016 (3)	−0.014 (3)	0.016 (3)
O1	0.066 (2)	0.046 (2)	0.051 (2)	0.0027 (18)	−0.0140 (18)	−0.0006 (17)
O2	0.073 (3)	0.036 (2)	0.062 (2)	0.0043 (17)	−0.012 (2)	−0.0011 (17)
O3	0.080 (3)	0.053 (2)	0.041 (2)	0.0143 (19)	−0.0046 (19)	0.0065 (17)
O4	0.069 (2)	0.055 (2)	0.044 (2)	0.0031 (19)	0.0036 (18)	−0.0063 (18)
O5	0.077 (3)	0.056 (3)	0.089 (3)	0.009 (2)	0.023 (2)	0.021 (2)
O6	0.069 (3)	0.047 (2)	0.086 (3)	0.005 (2)	0.011 (2)	0.010 (2)
O7	0.098 (4)	0.084 (3)	0.166 (5)	0.015 (3)	0.063 (3)	0.012 (3)
O8	0.089 (4)	0.157 (5)	0.093 (4)	−0.015 (4)	0.031 (3)	−0.047 (4)
O9	0.072 (3)	0.121 (4)	0.089 (4)	−0.004 (3)	0.006 (3)	−0.021 (3)
O10	0.089 (4)	0.160 (6)	0.150 (6)	−0.001 (4)	0.060 (4)	−0.004 (4)
O11	0.151 (4)	0.059 (3)	0.058 (3)	−0.038 (3)	−0.016 (3)	0.006 (2)
O12	0.099 (3)	0.066 (3)	0.061 (3)	−0.022 (2)	−0.019 (2)	0.012 (2)
O13	0.170 (5)	0.058 (3)	0.106 (4)	−0.032 (3)	−0.037 (4)	0.035 (3)
C19	0.093 (5)	0.061 (4)	0.079 (5)	0.007 (3)	−0.034 (4)	0.002 (3)
C2	0.055 (3)	0.042 (3)	0.058 (3)	−0.006 (2)	0.000 (3)	−0.012 (3)
C3	0.071 (4)	0.073 (4)	0.075 (4)	−0.005 (3)	−0.009 (3)	−0.018 (4)
C4	0.078 (5)	0.079 (5)	0.108 (6)	−0.018 (4)	0.001 (4)	−0.051 (4)
C5	0.060 (4)	0.052 (4)	0.139 (7)	0.000 (3)	0.003 (4)	−0.032 (4)
C6	0.050 (3)	0.049 (4)	0.090 (5)	−0.008 (3)	0.008 (3)	−0.018 (3)
C1	0.042 (3)	0.047 (3)	0.063 (4)	−0.001 (2)	0.002 (3)	−0.007 (3)
C7	0.067 (4)	0.034 (3)	0.162 (8)	0.000 (3)	−0.002 (5)	0.005 (4)
C8	0.125 (8)	0.042 (5)	0.129 (8)	0.015 (5)	0.024 (7)	0.035 (5)
C9	0.25 (3)	0.102 (12)	0.117 (12)	0.018 (12)	−0.056 (14)	0.049 (10)
C10	0.170 (11)	0.142 (10)	0.186 (11)	−0.007 (8)	0.030 (8)	−0.002 (8)
C8'	0.090 (10)	0.072 (9)	0.098 (9)	0.012 (8)	0.017 (8)	0.016 (7)
C9'	0.103 (11)	0.085 (9)	0.092 (9)	0.032 (9)	0.010 (8)	0.009 (9)
C10'	0.150 (16)	0.142 (16)	0.145 (16)	0.002 (10)	0.025 (10)	0.001 (10)
C11	0.082 (5)	0.121 (7)	0.068 (5)	0.039 (5)	0.014 (4)	0.043 (5)
C12	0.055 (3)	0.106 (6)	0.052 (4)	0.012 (4)	0.007 (3)	0.029 (4)
C13	0.070 (4)	0.144 (8)	0.047 (4)	0.008 (5)	−0.006 (3)	0.025 (5)
C14	0.076 (5)	0.165 (9)	0.037 (4)	−0.015 (5)	−0.001 (3)	0.006 (5)
C15	0.059 (4)	0.115 (6)	0.042 (4)	−0.013 (4)	0.005 (3)	−0.009 (4)
C16	0.048 (3)	0.071 (4)	0.047 (3)	−0.001 (3)	0.006 (3)	−0.003 (3)
C17	0.046 (3)	0.087 (5)	0.033 (3)	0.006 (3)	0.004 (2)	0.008 (3)
C18	0.084 (4)	0.067 (4)	0.065 (4)	0.010 (3)	0.009 (3)	−0.016 (3)
O14	0.068 (3)	0.082 (3)	0.088 (3)	−0.014 (2)	0.010 (2)	−0.017 (2)
C20	0.085 (5)	0.128 (7)	0.131 (7)	−0.005 (5)	0.046 (5)	−0.018 (6)
C21	0.063 (4)	0.058 (4)	0.086 (5)	−0.002 (3)	0.009 (4)	0.006 (3)
C22	0.074 (4)	0.098 (6)	0.096 (5)	−0.015 (4)	−0.005 (4)	−0.014 (4)

### Geometric parameters (Å, °)

**Table:**

Tb1—O2	2.353 (3)	C4—H2	0.9300
Tb1—O3	2.360 (3)	C5—C6	1.382 (8)
Tb1—O8	2.427 (5)	C5—H3	0.9300
Tb1—O6	2.436 (4)	C6—C1	1.419 (7)
Tb1—O9	2.446 (5)	C6—C7	1.441 (9)
Tb1—O5	2.472 (4)	C7—H4	0.9300
Tb1—O11	2.498 (4)	C8—C10	1.431 (7)
Tb1—O12	2.517 (4)	C8—C9	1.455 (7)
Tb1—O1	2.610 (3)	C8—H8	0.9800
Tb1—O4	2.616 (3)	C9—H9A	0.9700
Tb1—N3	2.871 (5)	C9—H9B	0.9700
Tb1—N4	2.875 (6)	C10—H10A	0.9600
Cu1—O3	1.895 (4)	C10—H10B	0.9600
Cu1—O2	1.901 (3)	C10—H10C	0.9600
Cu1—N1	1.903 (6)	C8'—C10'	1.40 (4)
Cu1—N2	1.929 (5)	C8'—C9'	1.457 (7)
N1—C7	1.265 (9)	C8'—H8'	0.9800
N1—C8'	1.490 (7)	C9'—H9'1	0.9700
N1—C8	1.504 (6)	C9'—H9'2	0.9700
N2—C11	1.251 (9)	C10'—H10D	0.9600
N2—C9'	1.469 (7)	C10'—H10E	0.9600
N2—C9	1.476 (7)	C10'—H10F	0.9600
N3—O7	1.208 (6)	C11—C12	1.438 (10)
N3—O5	1.234 (6)	C11—H5	0.9300
N3—O6	1.277 (5)	C12—C13	1.399 (9)
N4—O10	1.225 (7)	C12—C17	1.405 (7)
N4—O8	1.233 (7)	C13—C14	1.350 (9)
N4—O9	1.242 (7)	C13—H6	0.9300
N5—O13	1.211 (6)	C14—C15	1.364 (9)
N5—O12	1.237 (6)	C14—H7	0.9300
N5—O11	1.244 (6)	C15—C16	1.379 (7)
O1—C2	1.390 (6)	C15—H15	0.9300
O1—C19	1.440 (6)	C16—C17	1.385 (8)
O2—C1	1.326 (6)	C18—H18A	0.9600
O3—C17	1.346 (6)	C18—H18B	0.9600
O4—C16	1.396 (6)	C18—H18C	0.9600
O4—C18	1.437 (6)	O14—C21	1.217 (7)
C19—H1A	0.9600	C20—C21	1.495 (9)
C19—H1B	0.9600	C20—H20A	0.9600
C19—H1C	0.9600	C20—H20B	0.9600
C2—C3	1.368 (7)	C20—H20C	0.9600
C2—C1	1.397 (7)	C21—C22	1.471 (8)
C3—C4	1.408 (8)	C22—H22A	0.9600
C3—H1	0.9300	C22—H22B	0.9600
C4—C5	1.337 (9)	C22—H22C	0.9600
			
O2—Tb1—O3	63.73 (12)	C17—O3—Cu1	124.6 (3)
O2—Tb1—O8	73.33 (17)	C17—O3—Tb1	128.9 (3)
O3—Tb1—O8	98.85 (18)	Cu1—O3—Tb1	106.35 (15)
O2—Tb1—O6	117.86 (13)	C16—O4—C18	115.7 (4)
O3—Tb1—O6	119.11 (13)	C16—O4—Tb1	118.3 (3)
O8—Tb1—O6	141.76 (18)	C18—O4—Tb1	125.4 (3)
O2—Tb1—O9	101.25 (15)	N3—O5—Tb1	95.7 (3)
O3—Tb1—O9	74.70 (15)	N3—O6—Tb1	96.2 (3)
O8—Tb1—O9	50.48 (17)	N4—O8—Tb1	98.2 (4)
O6—Tb1—O9	140.80 (15)	N4—O9—Tb1	97.0 (4)
O2—Tb1—O5	75.17 (13)	N5—O11—Tb1	97.9 (3)
O3—Tb1—O5	75.56 (13)	N5—O12—Tb1	97.2 (3)
O8—Tb1—O5	146.97 (18)	O1—C19—H1A	109.5
O6—Tb1—O5	51.47 (13)	O1—C19—H1B	109.5
O9—Tb1—O5	148.17 (16)	H1A—C19—H1B	109.5
O2—Tb1—O11	121.96 (13)	O1—C19—H1C	109.5
O3—Tb1—O11	164.89 (15)	H1A—C19—H1C	109.5
O8—Tb1—O11	71.6 (2)	H1B—C19—H1C	109.5
O6—Tb1—O11	72.08 (15)	C3—C2—O1	124.6 (5)
O9—Tb1—O11	90.24 (17)	C3—C2—C1	121.6 (5)
O5—Tb1—O11	118.88 (16)	O1—C2—C1	113.8 (4)
O2—Tb1—O12	166.19 (14)	C2—C3—C4	118.1 (6)
O3—Tb1—O12	121.65 (13)	C2—C3—H1	120.9
O8—Tb1—O12	92.96 (18)	C4—C3—H1	120.9
O6—Tb1—O12	71.95 (14)	C5—C4—C3	120.6 (6)
O9—Tb1—O12	70.09 (16)	C5—C4—H2	119.7
O5—Tb1—O12	117.97 (15)	C3—C4—H2	119.7
O11—Tb1—O12	49.42 (13)	C4—C5—C6	123.0 (6)
O2—Tb1—O1	61.47 (11)	C4—C5—H3	118.5
O3—Tb1—O1	123.72 (12)	C6—C5—H3	118.5
O8—Tb1—O1	77.27 (16)	C5—C6—C1	117.2 (6)
O6—Tb1—O1	77.77 (12)	C5—C6—C7	119.8 (6)
O9—Tb1—O1	127.60 (15)	C1—C6—C7	123.0 (6)
O5—Tb1—O1	79.22 (13)	O2—C1—C2	116.3 (5)
O11—Tb1—O1	66.65 (12)	O2—C1—C6	124.5 (5)
O12—Tb1—O1	114.63 (12)	C2—C1—C6	119.2 (5)
O2—Tb1—O4	122.57 (12)	N1—C7—C6	126.7 (6)
O3—Tb1—O4	61.61 (12)	N1—C7—H4	116.6
O8—Tb1—O4	130.88 (16)	C6—C7—H4	116.6
O6—Tb1—O4	76.55 (13)	C10—C8—C9	102.5 (14)
O9—Tb1—O4	80.41 (15)	C10—C8—N1	118.4 (11)
O5—Tb1—O4	75.68 (13)	C9—C8—N1	114.4 (10)
O11—Tb1—O4	115.40 (13)	C10—C8—H8	106.9
O12—Tb1—O4	67.72 (12)	C9—C8—H8	106.9
O1—Tb1—O4	151.75 (11)	N1—C8—H8	106.9
O2—Tb1—N3	97.24 (13)	C8—C9—N2	101.0 (10)
O3—Tb1—N3	96.33 (14)	C8—C9—H9A	111.6
O8—Tb1—N3	156.01 (17)	N2—C9—H9A	111.6
O6—Tb1—N3	26.24 (12)	C8—C9—H9B	111.6
O9—Tb1—N3	152.87 (16)	N2—C9—H9B	111.6
O5—Tb1—N3	25.33 (13)	H9A—C9—H9B	109.4
O11—Tb1—N3	96.66 (16)	C10'—C8'—C9'	87.5 (18)
O12—Tb1—N3	94.76 (15)	C10'—C8'—N1	120 (2)
O1—Tb1—N3	78.85 (13)	C9'—C8'—N1	102.3 (19)
O4—Tb1—N3	72.91 (12)	C10'—C8'—H8'	114.4
O2—Tb1—N4	87.52 (15)	C9'—C8'—H8'	114.4
O3—Tb1—N4	87.12 (16)	N1—C8'—H8'	114.4
O8—Tb1—N4	25.11 (16)	C8'—C9'—N2	114.7 (19)
O6—Tb1—N4	148.99 (15)	C8'—C9'—H9'1	108.6
O9—Tb1—N4	25.39 (16)	N2—C9'—H9'1	108.6
O5—Tb1—N4	159.54 (15)	C8'—C9'—H9'2	108.6
O11—Tb1—N4	79.46 (18)	N2—C9'—H9'2	108.6
O12—Tb1—N4	80.33 (16)	H9'1—C9'—H9'2	107.6
O1—Tb1—N4	102.25 (17)	C8'—C10'—H10D	109.5
O4—Tb1—N4	105.80 (17)	C8'—C10'—H10E	109.5
N3—Tb1—N4	175.01 (15)	H10D—C10'—H10E	109.5
O3—Cu1—O2	81.88 (15)	C8'—C10'—H10F	109.5
O3—Cu1—N1	173.3 (2)	H10D—C10'—H10F	109.5
O2—Cu1—N1	96.5 (2)	H10E—C10'—H10F	109.5
O3—Cu1—N2	96.0 (2)	N2—C11—C12	127.3 (6)
O2—Cu1—N2	172.7 (2)	N2—C11—H5	116.4
N1—Cu1—N2	84.8 (3)	C12—C11—H5	116.4
O3—Cu1—Tb1	41.50 (10)	C13—C12—C17	117.5 (7)
O2—Cu1—Tb1	41.33 (11)	C13—C12—C11	118.9 (6)
N1—Cu1—Tb1	137.72 (17)	C17—C12—C11	123.6 (6)
N2—Cu1—Tb1	137.4 (2)	C14—C13—C12	121.8 (7)
C7—N1—C8'	118.8 (11)	C14—C13—H6	119.1
C7—N1—C8	126.7 (7)	C12—C13—H6	119.1
C8'—N1—C8	35.8 (9)	C13—C14—C15	120.9 (7)
C7—N1—Cu1	124.4 (5)	C13—C14—H7	119.5
C8'—N1—Cu1	110.4 (10)	C15—C14—H7	119.5
C8—N1—Cu1	108.2 (6)	C14—C15—C16	119.2 (7)
C11—N2—C9'	126.8 (13)	C14—C15—H15	120.4
C11—N2—C9	120.9 (8)	C16—C15—H15	120.4
C9'—N2—C9	12 (2)	C15—C16—C17	121.1 (6)
C11—N2—Cu1	124.1 (5)	C15—C16—O4	124.8 (6)
C9'—N2—Cu1	108.9 (12)	C17—C16—O4	114.1 (5)
C9—N2—Cu1	114.8 (7)	O3—C17—C16	116.5 (5)
O7—N3—O5	123.8 (5)	O3—C17—C12	124.0 (6)
O7—N3—O6	120.0 (5)	C16—C17—C12	119.4 (5)
O5—N3—O6	116.2 (4)	O4—C18—H18A	109.5
O7—N3—Tb1	174.9 (4)	O4—C18—H18B	109.5
O5—N3—Tb1	59.0 (3)	H18A—C18—H18B	109.5
O6—N3—Tb1	57.5 (3)	O4—C18—H18C	109.5
O10—N4—O8	123.5 (7)	H18A—C18—H18C	109.5
O10—N4—O9	122.3 (7)	H18B—C18—H18C	109.5
O8—N4—O9	114.2 (6)	C21—C20—H20A	109.5
O10—N4—Tb1	179.4 (5)	C21—C20—H20B	109.5
O8—N4—Tb1	56.7 (3)	H20A—C20—H20B	109.5
O9—N4—Tb1	57.6 (3)	C21—C20—H20C	109.5
O13—N5—O12	122.6 (5)	H20A—C20—H20C	109.5
O13—N5—O11	122.0 (5)	H20B—C20—H20C	109.5
O12—N5—O11	115.3 (5)	O14—C21—C22	120.9 (6)
O13—N5—Tb1	178.7 (5)	O14—C21—C20	121.1 (7)
O12—N5—Tb1	58.2 (3)	C22—C21—C20	117.9 (6)
O11—N5—Tb1	57.3 (3)	C21—C22—H22A	109.5
C2—O1—C19	116.9 (4)	C21—C22—H22B	109.5
C2—O1—Tb1	117.3 (3)	H22A—C22—H22B	109.5
C19—O1—Tb1	125.7 (3)	C21—C22—H22C	109.5
C1—O2—Cu1	124.4 (3)	H22A—C22—H22C	109.5
C1—O2—Tb1	128.4 (3)	H22B—C22—H22C	109.5
Cu1—O2—Tb1	106.42 (16)		

## References

[bb1] Higashi, T. (1995). *ABSCOR* Rigaku Corporation, Tokyo, Japan.

[bb2] Kara, H., Elerman, Y. & Prout, K. (2000). *Z. Naturforsch. Teil B*, **55**, 1131–1136.

[bb3] Rigaku (1998). *RAPID-AUTO* Rigaku Corporation, Tokyo, Japan.

[bb4] Rigaku/MSC (2002). *CrystalStructure* Rigaku/MSC Inc., The Woodlands, Texas, USA.

[bb5] Sheldrick, G. M. (2008). *Acta Cryst.* A**64**, 112–122. 10.1107/S0108767307043930 18156677

[bb6] Sun, W.-B., Gao, T., Yan, P.-F., Li, G.-M. & Hou, G.-F. (2007). *Acta Cryst.* E**63**, m2192.

